# 9-Ethyl-3-(imidazo[1,2-*a*]pyrimidin-3-yl)-9*H*-carbazole

**DOI:** 10.1107/S1600536808038300

**Published:** 2008-11-22

**Authors:** Ping-Hsin Huang, Guan-Ji Chen, Yuh-Sheng Wen

**Affiliations:** a Institute of Chemistry, Academia Sinica, Nankang, Taipei, Taiwan, and Cardinal Tien College of Healthcare and Management, Taipei, Taiwan; b Institute of Chemistry, Academia Sinica, Nankang, Taipei, Taiwan

## Abstract

The title compound, C_20_H_16_N_4_, is a precursor for the production of electron-transporting and -emitting materials. The bond lengths and angles in this compound are normal. In the crystal structure, there are no significant hydrogen-bonding inter­actions or π–π stacking inter­actions between mol­ecules.

## Related literature

For general background to the use of small organic molecules or organic polymers as electroluminescent materials, see: Burroughes *et al.* (1990 ▶); Tang & VanSlyke (1987 ▶).
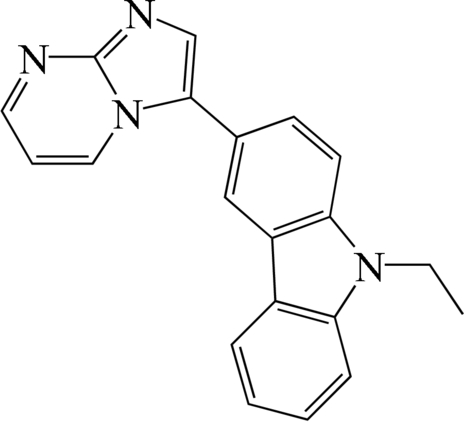


## Experimental

### 

#### Crystal data

C_20_H_16_N_4_
*M* *_r_* = 312.37
Monoclinic, 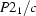

*a* = 13.9106 (3) Å
*b* = 9.3187 (2) Å
*c* = 12.9047 (3) Å
β = 112.712 (1)°
*V* = 1543.10 (6) Å^3^
*Z* = 4
Mo *K*α radiation
μ = 0.08 mm^−1^
*T* = 100.0 (1) K
0.36 × 0.32 × 0.28 mm

#### Data collection

Bruker SMART CCD area-detector diffractometer
Absorption correction: none
11748 measured reflections
2717 independent reflections
1991 reflections with *I* > 2σ(*I*)
*R*_int_ = 0.033

#### Refinement

*R*[*F* ^2^ > 2σ(*F* ^2^)] = 0.030
*wR*(*F* ^2^) = 0.072
*S* = 0.91
2717 reflections
218 parameters
H-atom parameters constrained
Δρ_max_ = 0.19 e Å^−3^
Δρ_min_ = −0.19 e Å^−3^

### 

Data collection: *SMART* (Bruker, 2001 ▶); cell refinement: *SAINT* (Bruker, 2001 ▶); data reduction: *SAINT*; program(s) used to solve structure: *SHELXS97* (Sheldrick, 2008 ▶); program(s) used to refine structure: *SHELXL97* (Sheldrick, 2008 ▶); molecular graphics: *ORTEP-3 for Windows* (Farrugia, 1997 ▶); software used to prepare material for publication: *WinGX* (Farrugia, 1999 ▶).

## Supplementary Material

Crystal structure: contains datablocks I, global. DOI: 10.1107/S1600536808038300/ww2131sup1.cif

Structure factors: contains datablocks I. DOI: 10.1107/S1600536808038300/ww2131Isup2.hkl

Additional supplementary materials:  crystallographic information; 3D view; checkCIF report

## Figures and Tables

**Fig. 1.**
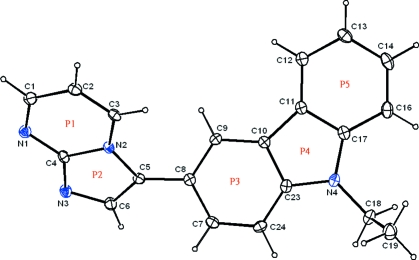
A molecular structure of (I) with 30% probability displacement ellipsoids, showing the atom-numbering scheme employed.

**Fig. 2.**
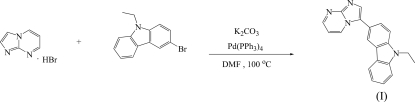
The formation of the title compound.

## supplementary crystallographic information

### Comment

The application of organic electroluminescent (OEL) in flatpanel displays using small organic molecules or organic polymers has been intensively pursued after the reports by Kodak's team (Tang & VanSlyke, 1987) and Cambridge's group (Burroughes *et al.*, 1990). The molecular structure of is shown in Fig. 1. The dihedral angle between the imidazole (P2) and phenyl ring of carbazole (P3) is 34.63 (8)°. Furthermore, the dihedral angles are 4.64 (8)°, 0.90 (8)° and 0.97 (8)° for P1/P2, P3/P4 and P4/P5, respectively.

### Experimental

The compound was synthesized by the following procedure. Imidazo[1,2-*a*]pyrimidine hydrobromide (2.0 g, 0.01 mol), 3-bromo-9-ethyl-9*H*-carbazole (4.6 g, 1.15 eq), Pd(PPh_3_)_4_ (0.23 g, 0.02 eq), K_2_CO_3_ (2.8 g, 2 eq), and *N*,*N*-dimethylformamide (5 ml) were charged in a two-necked flask kept under nitrogen. The mixture was heated to reflux for 48 h. After cooling, it was quenched with 5 ml of water. The solvent was removed under vacuum and the residue was extracted with dichloromethane/water. The organic layer was dried over MgSO_4_ and filtered. Evaporation of the solvent left a brown residue that was chromatographed through silica gel using dichloromethane/hexane (19:1) mixture as eluant. The compound was obtained as yellow solid in 37% yield. FW:312.4;FAB MS: m/e 313.3 (*M*^+^ + H). ^1^H NMR (CDCl_3_, δ/ ppm): 8.68 (dd, 1H, J = 6.8 Hz, J = 1.8 Hz), 8.57 (dd, 1H, J = 3.9 Hz, J = 1.9 Hz), 8.21 (s, 1H), 8.11 (d, 1H, J = 7.8 Hz), 7.92 (s, 1H), 7.59–7.49 (m, 3H), 7.45 (d, 1H, J = 8.1 Hz), 7.26(t, 1H, J = 7.3 Hz), 6.89 (dd, 1H, J = 6.8 Hz, J = 4.0 Hz).

### Refinement

H atoms were located geometrically and treated as riding atoms, with C—H = 0.93–0.96Å, and with *U*_iso_(H) = 1.2*U*_eq_(C).

### Crystal data

**Table:**

C_20_H_16_N_4_	*F*_000_ = 656
*M**_r_* = 312.37	*D*_x_ = 1.345 Mg m^−^^3^*D*_m_ = 1.345 Mg m^−^^3^*D*_m_ measured by not measured
Monoclinic, *P*2_1_/*c*	Mo *K*α radiation λ = 0.71073 Å
Hall symbol: -P 2ybc	Cell parameters from 3698 reflections
*a* = 13.9106 (3) Å	θ = 2.7–30.2º
*b* = 9.3187 (2) Å	µ = 0.08 mm^−^^1^
*c* = 12.9047 (3) Å	*T* = 100.0 (1) K
β = 112.712 (1)º	Prism, yellow
*V* = 1543.10 (6) Å^3^	0.36 × 0.32 × 0.28 mm
*Z* = 4	

### Data collection

**Table:**

Bruker SMART CCD area-detector diffractometer	1991 reflections with *I* > 2σ(*I*)
Radiation source: fine-focus sealed tube	*R*_int_ = 0.033
Monochromator: graphite	θ_max_ = 25.0º
*T* = 100.0(1) K	θ_min_ = 1.6º
ω and φ scans	*h* = −16→16
Absorption correction: none	*k* = −11→11
11748 measured reflections	*l* = −15→15
2717 independent reflections	

### Refinement

**Table:**

Refinement on *F*^2^	Hydrogen site location: inferred from neighbouring sites
Least-squares matrix: full	H-atom parameters constrained
*R*[*F*^2^ > 2σ(*F*^2^)] = 0.030	*w* = 1/[σ^2^(*F*_o_^2^) + (0.0416*P*)^2^] where *P* = (*F*_o_^2^ + 2*F*_c_^2^)/3
*wR*(*F*^2^) = 0.072	(Δ/σ)_max_ = 0.001
*S* = 0.91	Δρ_max_ = 0.19 e Å^−^^3^
2717 reflections	Δρ_min_ = −0.18 e Å^−^^3^
218 parameters	Extinction correction: SHELXL97 (Sheldrick, 2008), Fc^*^=kFc[1+0.001xFc^2^λ^3^/sin(2θ)]^-1/4^
Primary atom site location: structure-invariant direct methods	Extinction coefficient: 0.0063 (8)
Secondary atom site location: difference Fourier map	

### Special details

**Table:**

Geometry. All e.s.d.'s (except the e.s.d. in the dihedral angle between two l.s. planes) are estimated using the full covariance matrix. The cell e.s.d.'s are taken into account individually in the estimation of e.s.d.'s in distances, angles and torsion angles; correlations between e.s.d.'s in cell parameters are only used when they are defined by crystal symmetry. An approximate (isotropic) treatment of cell e.s.d.'s is used for estimating e.s.d.'s involving l.s. planes.
Refinement. Refinement of *F*^2^ against ALL reflections. The weighted *R*-factor *wR* and goodness of fit *S* are based on *F*^2^, conventional *R*-factors *R* are based on *F*, with *F* set to zero for negative *F*^2^. The threshold expression of *F*^2^ > σ(*F*^2^) is used only for calculating *R*-factors(gt) *etc*. and is not relevant to the choice of reflections for refinement. *R*-factors based on *F*^2^ are statistically about twice as large as those based on *F*, and *R*- factors based on ALL data will be even larger.

### Fractional atomic coordinates and isotropic or equivalent isotropic displacement parameters (Å^2^)

**Table:**

	*x*	*y*	*z*	*U*_iso_*/*U*_eq_	
N4	1.35147 (8)	0.58921 (11)	0.45030 (9)	0.0200 (3)	
N3	0.96458 (8)	0.19984 (11)	0.66711 (9)	0.0225 (3)	
N1	0.85050 (8)	0.05126 (11)	0.51971 (9)	0.0209 (3)	
N2	0.98239 (8)	0.20433 (11)	0.50125 (8)	0.0166 (3)	
C17	1.37034 (10)	0.48850 (14)	0.38158 (10)	0.0196 (3)	
C16	1.43492 (10)	0.49764 (15)	0.32235 (11)	0.0256 (3)	
H16	1.4741	0.5797	0.3258	0.031*	
C14	1.43902 (10)	0.38125 (15)	0.25828 (11)	0.0279 (4)	
H14	1.4817	0.3852	0.2179	0.033*	
C13	1.38043 (10)	0.25737 (15)	0.25257 (11)	0.0253 (3)	
H13	1.3845	0.1805	0.2085	0.030*	
C12	1.31683 (10)	0.24836 (14)	0.31163 (11)	0.0210 (3)	
H12	1.2784	0.1656	0.3081	0.025*	
C11	1.31059 (9)	0.36437 (13)	0.37670 (10)	0.0179 (3)	
C10	1.25171 (9)	0.39349 (13)	0.44510 (10)	0.0166 (3)	
C9	1.17866 (9)	0.31495 (13)	0.47090 (10)	0.0171 (3)	
H9	1.1602	0.2230	0.4422	0.021*	
C8	1.13349 (9)	0.37494 (13)	0.53988 (10)	0.0171 (3)	
C7	1.16524 (10)	0.51203 (13)	0.58548 (10)	0.0195 (3)	
H7	1.1364	0.5503	0.6336	0.023*	
C24	1.23750 (10)	0.59182 (14)	0.56155 (10)	0.0203 (3)	
H24	1.2575	0.6824	0.5927	0.024*	
C23	1.27953 (9)	0.53254 (13)	0.48938 (10)	0.0180 (3)	
C18	1.39561 (10)	0.73286 (13)	0.47180 (11)	0.0248 (3)	
H18A	1.4685	0.7281	0.4827	0.030*	
H18B	1.3919	0.7690	0.5406	0.030*	
C19	1.33998 (11)	0.83644 (15)	0.37712 (12)	0.0305 (4)	
H19A	1.3718	0.9294	0.3954	0.046*	
H19B	1.2680	0.8430	0.3668	0.046*	
H19C	1.3449	0.8025	0.3091	0.046*	
C6	1.04213 (10)	0.29149 (14)	0.66997 (11)	0.0223 (3)	
H6	1.0810	0.3451	0.7331	0.027*	
C4	0.92857 (10)	0.14710 (13)	0.56400 (10)	0.0181 (3)	
C1	0.82339 (10)	0.02090 (13)	0.41227 (11)	0.0214 (3)	
H1	0.7709	−0.0464	0.3802	0.026*	
C2	0.86906 (10)	0.08412 (13)	0.34325 (11)	0.0205 (3)	
H2	0.8445	0.0624	0.2671	0.025*	
C3	0.94900 (10)	0.17667 (13)	0.38901 (10)	0.0184 (3)	
H3	0.9804	0.2203	0.3453	0.022*	
C5	1.05729 (10)	0.29722 (13)	0.57133 (10)	0.0173 (3)	

### Atomic displacement parameters (Å^2^)

**Table:**

	*U*^11^	*U*^22^	*U*^33^	*U*^12^	*U*^13^	*U*^23^
N4	0.0182 (6)	0.0194 (6)	0.0212 (6)	−0.0032 (5)	0.0064 (5)	0.0020 (5)
N3	0.0221 (6)	0.0286 (7)	0.0179 (6)	−0.0029 (5)	0.0090 (5)	−0.0014 (5)
N1	0.0186 (6)	0.0214 (6)	0.0225 (7)	−0.0001 (5)	0.0076 (5)	0.0002 (5)
N2	0.0167 (6)	0.0193 (6)	0.0148 (6)	0.0011 (5)	0.0071 (5)	0.0008 (5)
C17	0.0145 (7)	0.0257 (8)	0.0166 (7)	0.0018 (6)	0.0038 (6)	0.0037 (6)
C16	0.0167 (7)	0.0315 (8)	0.0279 (8)	−0.0014 (6)	0.0077 (7)	0.0065 (7)
C14	0.0189 (8)	0.0413 (9)	0.0273 (8)	0.0068 (7)	0.0131 (7)	0.0075 (7)
C13	0.0199 (8)	0.0338 (9)	0.0225 (8)	0.0065 (7)	0.0087 (6)	0.0006 (7)
C12	0.0164 (7)	0.0244 (8)	0.0205 (7)	0.0019 (6)	0.0054 (6)	0.0029 (6)
C11	0.0138 (7)	0.0219 (7)	0.0161 (7)	0.0021 (6)	0.0038 (6)	0.0032 (6)
C10	0.0151 (7)	0.0194 (7)	0.0130 (7)	0.0022 (6)	0.0030 (6)	0.0023 (6)
C9	0.0178 (7)	0.0164 (7)	0.0145 (7)	−0.0006 (6)	0.0033 (6)	0.0006 (6)
C8	0.0159 (7)	0.0206 (7)	0.0129 (7)	0.0015 (6)	0.0034 (6)	0.0021 (6)
C7	0.0207 (7)	0.0228 (7)	0.0146 (7)	0.0040 (6)	0.0065 (6)	0.0006 (6)
C24	0.0219 (7)	0.0167 (7)	0.0181 (7)	0.0001 (6)	0.0032 (6)	−0.0008 (6)
C23	0.0157 (7)	0.0200 (7)	0.0158 (7)	0.0007 (6)	0.0033 (6)	0.0050 (6)
C18	0.0229 (8)	0.0214 (8)	0.0284 (8)	−0.0050 (6)	0.0081 (6)	0.0026 (6)
C19	0.0288 (9)	0.0284 (8)	0.0367 (9)	0.0029 (7)	0.0152 (7)	0.0085 (7)
C6	0.0211 (7)	0.0286 (8)	0.0171 (7)	−0.0035 (6)	0.0072 (6)	−0.0041 (6)
C4	0.0184 (7)	0.0213 (7)	0.0166 (7)	0.0019 (6)	0.0090 (6)	0.0031 (6)
C1	0.0180 (7)	0.0207 (7)	0.0229 (8)	0.0011 (6)	0.0051 (6)	−0.0020 (6)
C2	0.0192 (7)	0.0238 (8)	0.0165 (7)	0.0020 (6)	0.0048 (6)	−0.0021 (6)
C3	0.0188 (7)	0.0226 (8)	0.0141 (7)	0.0045 (6)	0.0068 (6)	0.0013 (6)
C5	0.0160 (7)	0.0193 (7)	0.0157 (7)	0.0008 (6)	0.0049 (6)	−0.0014 (6)

### Geometric parameters (Å, °)

**Table:**

N4—C17	1.3841 (16)	C10—C23	1.4088 (17)
N4—C23	1.3871 (15)	C9—C8	1.3898 (16)
N4—C18	1.4541 (15)	C9—H9	0.9300
N3—C4	1.3222 (16)	C8—C7	1.4045 (17)
N3—C6	1.3652 (16)	C8—C5	1.4646 (17)
N1—C1	1.3194 (15)	C7—C24	1.3778 (17)
N1—C4	1.3508 (16)	C7—H7	0.9300
N2—C3	1.3640 (15)	C24—C23	1.3911 (17)
N2—C5	1.3874 (15)	C24—H24	0.9300
N2—C4	1.4031 (15)	C18—C19	1.5143 (18)
C17—C16	1.3889 (17)	C18—H18A	0.9700
C17—C11	1.4115 (17)	C18—H18B	0.9700
C16—C14	1.3782 (18)	C19—H19A	0.9600
C16—H16	0.9300	C19—H19B	0.9600
C14—C13	1.3986 (18)	C19—H19C	0.9600
C14—H14	0.9300	C6—C5	1.3693 (16)
C13—C12	1.3752 (17)	C6—H6	0.9300
C13—H13	0.9300	C1—C2	1.4074 (17)
C12—C11	1.3920 (17)	C1—H1	0.9300
C12—H12	0.9300	C2—C3	1.3499 (18)
C11—C10	1.4429 (16)	C2—H2	0.9300
C10—C9	1.3929 (16)	C3—H3	0.9300
			
C17—N4—C23	108.46 (10)	C8—C7—H7	118.8
C17—N4—C18	125.16 (10)	C7—C24—C23	117.87 (12)
C23—N4—C18	126.29 (11)	C7—C24—H24	121.1
C4—N3—C6	104.37 (10)	C23—C24—H24	121.1
C1—N1—C4	116.31 (11)	N4—C23—C24	129.87 (12)
C3—N2—C5	132.23 (10)	N4—C23—C10	109.06 (10)
C3—N2—C4	120.15 (11)	C24—C23—C10	121.07 (11)
C5—N2—C4	107.14 (10)	N4—C18—C19	112.72 (11)
N4—C17—C16	129.35 (12)	N4—C18—H18A	109.0
N4—C17—C11	109.28 (10)	C19—C18—H18A	109.0
C16—C17—C11	121.37 (12)	N4—C18—H18B	109.0
C14—C16—C17	117.86 (13)	C19—C18—H18B	109.0
C14—C16—H16	121.1	H18A—C18—H18B	107.8
C17—C16—H16	121.1	C18—C19—H19A	109.5
C16—C14—C13	121.53 (13)	C18—C19—H19B	109.5
C16—C14—H14	119.2	H19A—C19—H19B	109.5
C13—C14—H14	119.2	C18—C19—H19C	109.5
C12—C13—C14	120.51 (13)	H19A—C19—H19C	109.5
C12—C13—H13	119.7	H19B—C19—H19C	109.5
C14—C13—H13	119.7	N3—C6—C5	113.76 (11)
C13—C12—C11	119.36 (12)	N3—C6—H6	123.1
C13—C12—H12	120.3	C5—C6—H6	123.1
C11—C12—H12	120.3	N3—C4—N1	127.00 (11)
C12—C11—C17	119.38 (11)	N3—C4—N2	111.12 (11)
C12—C11—C10	134.22 (12)	N1—C4—N2	121.88 (11)
C17—C11—C10	106.39 (11)	N1—C1—C2	124.03 (12)
C9—C10—C23	119.86 (11)	N1—C1—H1	118.0
C9—C10—C11	133.33 (12)	C2—C1—H1	118.0
C23—C10—C11	106.80 (10)	C3—C2—C1	119.18 (12)
C8—C9—C10	119.59 (12)	C3—C2—H2	120.4
C8—C9—H9	120.2	C1—C2—H2	120.4
C10—C9—H9	120.2	C2—C3—N2	118.06 (12)
C9—C8—C7	119.17 (12)	C2—C3—H3	121.0
C9—C8—C5	122.22 (11)	N2—C3—H3	121.0
C7—C8—C5	118.54 (11)	C6—C5—N2	103.61 (10)
C24—C7—C8	122.38 (12)	C6—C5—C8	131.65 (12)
C24—C7—H7	118.8	N2—C5—C8	124.72 (11)
